# Practical Medicine

**Published:** 1851-11

**Authors:** Theodore S. Bell


ART. III.—*Practical Medicine.* By THEODORE S. BELL, M. D.

In the 13th number of Ranking's Half-Yearly Abstract of the Medical Sciences, a description of a "new epidemic exanthem" by Dr. Laycock, is quoted from the Medical Times, of March 8th, 1851. There has been a great number of cases of a similar disease in this city, during the past summer and the present autumn. The following is Dr. Laycock's description of this epidemic:

"The disease is mainly characterized by a succession of boils on various parts of the body, of various sizes, from a bean to a walnut. First, there is a small hard pimple, with, perhaps, a vesicle or circlet of vesicles on the top. This itches; the top is scratched off; when it is found that there is a small tumour in or below the derma, which becomes larger, inflamed, very painful, and at last suppurates, with an erysipelatous blush about it, and in bad cases with phlyctænæ. A number of these occur in succession on various parts of the body, but principally on the fore-arm, leg, and nates. Occasionally there is a vesicle only, which quickly puts on the appearance of ecthyma; and in one case at the Dispensary (in a child), there was just one large livid-looking phlyctæna, as large as a crown-piece, a solitary monster, of a startling aspect, so like gangrene it looked. Sometimes there is a solitary boil, large, angry-looking, and mischievous as a carbuncle. An aged lady, who came under my notice, had one of these on the mons veneris; and sometimes even the minor specimens are not to be distinguished from carbuncles. Very often after they have sloughed and healed they leave an indurated condition of the skin and subcutaneous cellular tissue, of a very unpleasant kind.

"The eruption, whatever form it may assume, has a definite period of duration, and continues for from two to six weeks. The furuncular form is not always more chronic than the ecthymatous, but for the most part it is so; the exceptions being those cases in which the patient is cachectic. It is not, however, as I have previously remarked, dependent at all upon a cachectic condition, for I have seen it in robust men and in very healthy children. Nevertheless, the cachectic suffer more from the disease, and perhaps they suffer also in greater numbers; but of this I have no certain information. In some instances you may clearly trace the localization of the boils to some local cause; for example, a crop will break out round a blister, or round another boil, if it be poulticed much, or round a burn. I have a case in which they have occurred on the neck, thorax, and upper arm of a young woman who has irritated her throat by the inunction of iodide of pottassium ointment; and another, in which a chronic psoriasis seems to have been the exciting cause. I suspect that any local irritation of the skin is sufficient to induce the disease in an individual within the sphere of the epidemic."

This is an accurate picture of numbers of cases that have occurred here this season. In connection with the tendency to this exanthem, there was an unusual number of those boils, commonly called catarrhs, underneath the fascia of the hands. There were eleven cases of this kind, under the observation of the writer, in one day.

Dr. Laycock details the exanthem he describes, under the following forms:

"1. There may be a solitary boil; such was the case of an old laborer who came from the country to the Dispensary, a week or two ago, with a large boil on the point of the elbow, phlyctænoid, and presenting inflammation of an erysipelatous character, extending down the arm to the wrist, and upwards towards the shoulder. 2. A solitary phlyctæna, or several. 3. Several boils, varying in size and character up to carbuncles, without any other cutaneous disease. 4. Boils, with ecthyma, eczema, or impetigo; but much more frequently with ecthyma. 5. The boils differ, in leaving or not leaving behind them an induration of the skin and subcutaneous tissue. How long the disease has been epidemic is uncertain. I certainly observed it more than eighteen months ago. Its progress is slow, for the inmates of a house do not suffer from it in rapid succession, so that only one or two in a family are affected at one time, except where the family is large. Out of sixty or seventy inmates of the private asylum in which I first saw it, there were not more than 10 per cent. affected at once; and in some the disease was so trifling, that it would not have been noticed under ordinary circumstances—perhaps one or two small boils and no more."

Dr. Laycock discusses the subject of epizootic contagious fevers, with a great deal of acumen, and leans to the opinion that the furuncular epidemic he describes may have its origin in an epizootic disease, and having thus originated, it may be transmitted from one individual to another. The entire subject is a very curious one, and deserves a great deal of close and nice observation.—Much obscurity reigns now over the whole matter of connection between epizootic diseases and certain diseases among men, possessing some of the features of the epizootic disease, while other symptoms show modifications that are perplexing. The discovery of the power of the vaccine virus, by Jenner, called forth a multitude of facts and reasonings on epizootic diseases, and their transmissibility to man. The subject was discussed with great ability and learning, voluminous tomes and multitudes of essays appeared, but now, after the lapse of fifty years, the subject is almost, if not quite as unsettled, as when the controversy commenced. This is not the place, however, for a disquisition on the curious, recondite and obscure questions referred to, and the matter is noticed here in order to direct the attention of medical men to the possible connection between some epizootic contagious disorders, and the exanthem described by Dr. Laycock.

In the course of Dr. Laycock's disquisition, he states a curious coincidence in puerperal disease. Dr. Laycock saw a modest, unmarried girl, with a disease resembling puerperal fever. The physician in attendance on the case, with whom Dr. Laycock was in consultation, had two parturient females under his care, while in attendance on this girl, and in a day or two after the death of the girl, these ladies were attacked with puerperal fever of a very acute type. They died in a few hours after the commencement of the attack. By investigation it was discovered, that the unmarried girl, who was the first victim, had been in close attendance on a cow which died of milk fever, "in a shed almost within the house."

The treatment recommended by Dr. Laycock, for what he calls a "new exanthem," is such as was found most successful in this city. The following is Dr. Laycock's detail of his management of the disease:

"In treating this furuncular disease, you have little more to do, in ordinary cases, than to let it run its course, which is completed in three or four weeks. An occasional purgative and warm bath will be useful in allaying inflammatory action, and perhaps diminishing the number of boils. Two grains of calomel with rhubarb, colocynth, or scammony, twice a week, will be useful; in the more severe cases, the mineral acids and vegetable bitters must be added, a good diet; the liquor of amorphous sulphate of quinine in full doses with dilute nitric acid, has been found useful. In all cases, however, purgatives and the warm bath are beneficial. I do not think you do much good by direct applications to the boils,—as cataplasms, fomentations, irritating plasters, &c. So soon as suppuration is established, a free incision should be made into the boil, and then the water-dressing applied: care should be taken that the pus do not flow on the adjoining skin, and (as in erysipelas) that the sponges, &c., be not used by other patients or persons."

*Remedy for Deficient Lactation.*—The *Stethoscope* reports the testimony of Dr. Deane, of Virginia, in favor of the efficacy of the leaves of the castor oil bean in the removal of deficient lactation. Dr. Deane's case was one of some interest. A strong decoction of the leaves, as a ptisan, and a poultice of the leaves to the breasts, were successful in inducing a plentiful supply of milk in about twenty-four hours after their application commenced. Castor oil is known to be quite a popular purgative in the puerperal condition, and some portion of its popularity may be due to its utility in aiding lactation.

*Yeast in Malignant Scarlet Fever.*—The use of yeast in low forms of fever, dysentery, etc., has been commended several times in this Journal. It is, in its proper place, an invaluable remedy. In the *Medical Gazette*, January 10th, 1851, Mr. Bennett, of Gateshead, says: "After ammonia, the mineral acids, chlorate of potash, etc., have failed, and the application of nitrate of silver besides, one or two tablespoonfuls of fresh yeast *frequently given* (according to the age and malignancy of the case) has, in my practice at least, been quickly efficacious as an antiseptic and stimulant." No one who has had opportunities of seeing the effects of yeast over such conditions of the constitution as Dr. Bennett describes can doubt the value of the remedy. Many years ago, the writer saw this remedial agent described in an old English magazine, by an Episcopal clergyman, in its remarkable efficacy in a typhus epidemic that had been very fatal up to the time the yeast was tried. The good effects were at once remarkable, and I have seen them abundantly confirmed in a great variety of cases, in the past few years.

*The Treatment of Small-Pox.*—Mr. J. G. Pasquin, M. R.C.L.S., of Birmingham, publishes an interesting communication in the *London Lancet*, on the treatment of small-pox. The treatment recommended by Mr. Pasquin is very judicious, and deserves attention. Mr. Pasquin says:

"After mature deliberation thereon, it was my opinion that the pitting and consequent disfiguration of the face after that disorder, was dependent upon the confinement of the matter in the pocks for too great a length of time, which thereby would cause a slough to form in the cellular tissue lying between the cuticle and fascia of the face, which, being so thin, is never more regenerated, thereby causing the cuticle to fall into the space where the cellular tissue is then wanting, and thus follows the pitting.

"Secondly. I was of opinion, that by puncturing each pock previously to its coming to perfection, and then treating it with poultice, as a common abscess, I should not only avoid the pitting but also draw out of the system that putrid matter which, had the pock been left to ripen, would not only have caused the slough and pitting, but would have been absorbed into the body, and produced most injurious results to the system in general.

"Thirdly. In numerous instances, I have seen patients die from the eruption breaking out, not only on the tongue, fauces, and pharynx, but also on the most delicate part, the larynx. This I also thought might be obviated by placing a few leeches over the external region of the larynx, supposing it would, by diminishing the circulation in that region, reduce the size of the pocks, and also give play to the thyro-arytenoid muscle, and thereby prevent suffocation.

"Having formed these opinions, I felt determined to try them in the next case of small-pox that came under my notice, which I have now done, and beg leave to report to you the result of my experience:—I have had seven cases, four wherein the larynx was not at all affected, on which I tried the experiment of puncturing every pock on the face, and afterwards applying repeated poultices. This treatment succeeded to my utmost satisfaction, the face being left as clear of marks as it was previously to the attack of small-pox. I had three more with affection of the larynx, the respiration being so difficult that I expected asphyxia would come on in a few hours. To these I applied leeches over the region of the larynx, and on the following morning, I found that the respiration had become perfectly free and easy."

*A peculiar appearance in the Gums of Consumptive Patients.*—Dr. T. Thompson, Physician to the Hospital for Consumption, details, in the Proceedings of the Royal Medical and Chirurgical Society, a singular appearance in the gums of consumptive patients. These remarks, coming from one who enjoys such means of observation as those possessed by Dr. Thompson, are calculated to arouse the attention of the profession, and it is the duty of all who enjoy the means of doing so, either to refute or confirm the statements of Dr. Thompson. The Lancet says:

"The author, after glancing at the risk of allowing the study of auscultation to supersede attention to general symptoms, refers to the discovery, communicated to the Society by the late Dr. Burton, of a blue line at the edge of the gums, as indicative of the presence of lead, and thence derives an encouragement to the examination of this part of the system, when there is reason to suspect any poisoned or morbid condition of the blood. He then presents the results of his observations in reference to this inquiry in cases of consumption, and avows his conviction of the frequent existence, in phthisical subjects, of a mark at the reflected edge of the gums, deeper in color than the adjoining surface; in some patients a mere streak on a raised border, in others, a margin more than a line in breadth, of a vermilion tint, inclining to lake: the mark being most distinct around the lower incisors, but usually observable in both jaws, and often around the molar, but modified in its situation by the form of the mouth. The author has examined some hundred cases in the course of the investigation, and gives the analysis of 102, of whom he has full records. In forty of forty-eight women the gingival margin is present; and in fifty-four phthisical men, although in a few the line is so faint as to be open to question, there is only one in whom it can be considered as decidedly absent. He has reasons for suspecting that the same condition of the system which produces this state of the gums tends also to produce clubbing of the fingers; but he considers that the change in the extremity of the fingers rarely occurs till sometime after the streak is manifest in the gums. Of seventy-six patients, forty-five were found to have clubbed fingers; of these forty-five only one had gums free from the characteristic margin; yet twenty of the seventy-six had marginated gums but no expansion of the extremities of the fingers.

"The author discusses the effect of various modifying influences, such as hereditary tendency, catamenial disturbances, and habits as respects cleanliness, but cannot connect the presence of the symptoms in question with any of these circumstances; but he is of opinion that causes which irritate the mucous membrane tend to accelerate and increase the manifestations of the margin. He suggests this as an explanation of the more frequent absence of the line in women than in men, and dwells on its practical importance, as indicating, in such cases, the use of refrigerants, as preliminary to the introduction of tonic remedies. The author canvasses the question whether a similar line exists in any other disease; he allows that M. Fredericq may be correct in the opinion that certain changes in the gums occur towards the close of various chronic diseases, but he has never yet observed the peculiar margin described in this communication, without detecting other indications of consumption, although frequently only incipient. As respects prognosis in phthisis, he proposes the general rule, that cases in which the streak is observed early, or is broad or deep colored, tend to proceed more rapidly than those in which it is absent or slight; whilst freedom from the streak, even in the third stage, affords encouragement in treatment.

"In reference to diagnosis, the author believes,—1st. That the absence of the streak in men affected with inconclusive symptoms of phthisis, may incline us to a favorable interpretation of any such suspicious indications; but that in women, rather less weight is to be attributed to this negative sign. 2d. That the presence of the sign in women is almost conclusive evidence of the presence of the tubercular element in the blood.

"The paper concludes with the remark, that the symptom therein described is one of many proofs that consumption is *not* exclusively a local disease, but rather a constitutional condition, requiring for its elucidation and treatment far more than an acquaintance, however exact, with the phenomena of auscultation."

*Effects of Aneurism.*—Dr. Banks, Queen's Professor of the Practice of Medicine to the School of Physic in Ireland, reports a series of interesting cases in the August number of the Dublin Quarterly Journal of Medical Science. In this series is a case of a peculiar form of Aphonia, and it possesses so many interesting features that are valuable in diagnosis, that no apology is necessary for entering into the details of the case. The writer has a patient now under his care, who is the victim probably of the form of disease described by Dr. Banks. Dr. Banks reports:

"The next case which I propose to place on record is one of *Aneurism,* which, from circumstances connected with it, I consider to be of surpassing interest. The man who was the subject of it was long under my observation, first at the Whitworth, and subsequently at Sir Patrick Dun's, Hospital. I watched the case very closely, and I was most anxious to have the power of comparing the symptoms observed during life with the morbid appearances. A fortnight before his death, however, he passed from under my care, and went to the country, thinking himself equal to engage in some easy work, but in a week he returned to Dublin, and was admitted into Steevens' hospital, under Dr. Croker, who kindly permitted me to follow up the case to its fatal termination.

"At Steevens' hospital the patient was under the notice of Dr. Robert Macdonnell, who had carefully and accurately reported his case for me as follows, while acting as clinical clerk in the Whitworth and Sir Patrick Dun's Hospitals.

"Thomas Reddy, aged 40 years, by trade a tailor, was admitted into the Whitworth Hospital, under Dr. Banks' care, in June, 1850, when he stated that for two months previously he had been suffering from the effects of a severe cold, attended with unceasing cough, and a peculiar hoarseness of voice; on account of this, the uvula, which was much elongated and relaxed, was removed, and he was then discharged.

"He next came under my observation whilst under Dr. Law's care, in Sir Patrick Dun's Hospital, in December of the same year. He now stated that ever since the removal of his uvula his voice has gradually become more affected; at present the modification of it is very remarkable, and not very easily described: he speaks in a loud blowing whisper, is unable to articulate more than a few words at a time without taking a deep inspiration, and he has constant cough. Respiration is compartively feeble in the left lung; the sounds of the heart are healthy; but to the right of the sternum, and at a point corresponding to the interval between the cartilages of the second and third ribs, there is an impulse almost as strong as over the heart itself; careful percussion with the pleximeter detects slight dulness in the same locality; and on the left side, in the carotid artery, low down in the neck, a very feeble bruit is audible, accompanying the first sound of the heart. On strict inquiry, it appears that some months ago he suffered from dysphagia, solid food, however, causing him comparatively little uneasiness, while he found much difficulty in swallowing liquids: this symptom, together with pains in his neck and left shoulder, which he then suffered from also, has now entirely disappeared. He left the hospital, according to his own wish, December 29, 1850.

"He was readmitted into Sir Patrick Dun's Hospital, under Dr. Banks' care, on February 26, 1851, at which time the reporter (then acting as Dr. Banks' clinical clerk) took the following note of his case:—The striking peculiarity of voice already observed is the first symptom that attracts attention; it is still of the same curious blowing character—a loud whisper, like a person trying to speak above his breath, but unable to do so; the cough, too, is of somewhat the same nature: to use his own words, he 'never can get a hold of it.' The sputa consist of thick, viscid, muco-purulent matter, expectorated with much difficulty, and in large quantity. He is not unfrequently awakened at night, and obliged to start up in bed, from paroxysms of dyspnœa. The pulse at the left wrist is very weak; and although, on percussion, both lungs appear equally resonant posteriorly, yet respiration in the left is extremely feeble, while it is puerile all over the right side. The sounds of the heart are healthy, except that the first seems somewhat lengthened; a strong impulse still exists to the right of the sternum, at the point already indicated; there is marked and extensive dulness under the left clavicle, about two inches beneath the center of which, a bruit, synchronous with the second sound of the heart, is distinctly audible. There is greater fulness in the left clavicular region than in the right; and the veins of the neck and left arm are very much enlarged. He complains of incessant thirst. The patient left the hospital, of his own accord, on April 28th.

"The patient came under the observation of the reporter, for the third time, in Steevens' Hospital, where he was admitted, under Dr. Crocker's care, on May 8th, in an almost dying state. On careful examination of the chest, none of the stethoscopic signs of aneurism were now to be detected; no bruit was audible, either over the heart, or in the region where, from former examination, it is certain that an aneurism is situated. The entire left side is dull, and no respiration is to be heard in any part of that lung. The voice and cough (the latter of which never ceases now) are still of the same curious character; there are spots of purpura on the extremities; and there is a very abundant expectoration. An attack of diarrhea, which came on some days before his admission, proving uncontrollable, terminated his existence, on May 15, 1851.

"*Post-Mortem Examination, twelve hours after death.* Great emaciation; head not examined. On opening the thorax there was discovered an immense dilatation of the sinus of the aorta, and an aneurism, nearly the size of a cocoa nut, engaged that part of the arch of the aorta, from which the left carotid and subclavian arteries arise; it pressed backwards on the œsophagus, but was for the most part imbedded in the upper part of the left lung, on the root of which it impinged; the vena innominata of the left side passed directly across the tumor, and the pneumogastic nerve of the same side was actually flattened out over it, and appeared larger than its fellow; the phrenic nerve also passed across the aneurism. The left lung was universally adherent, except near the base behind, where there existed, amid old adhesions, a local empyema, containing about eight ounces of pus; it was in a cirrhosed condition, tough and gristly, having its pleural investment and the septa derived from this covering much thickened; it was quite riddled with small abscesses, varying in size from that of a walnut to that of a pea. The right lung was healthy. Extensive ulceration was discovered in the large intestine.

"The symptoms during life having directed especial attention to the larynx, a very careful examination which was made of it disclosed the following pathological facts: All the muscles of the larynx, which are undoubtedly supplied by the recurrent laryngeal nerve, were completely atrophied on the left side; indeed the latter crico-arytenoid and the thyro-arytenoid had disappeared on that side. While in the recent state the contrast between the right and left posterior crico-arytenoids was very striking; the former was a well-developed, bright-red colored muscle, while the left had almost entirely degenerated into a pale cellular structure, having little appearance of muscle left. The oblique fibres of the arytenoid muscle seemed also to have undergone a similar degeneration, particularly those passing from the base of the left to the apex of the right arytenoid cartilage; the transverse fibres, however, of this muscle, were very fully developed, and well colored. As was to be expected, neither of the crico-thyroid muscles were at all altered in structure.

"It is not my intention to dwell at length upon the particulars of this case, but I wish to direct attention to the vocal phenomena, and their connexion with the condition of the muscles of the larynx. The laryngeal symptoms in certain cases of thoracic aneurism, so frequently simulating disease of the larynx, have been over and over again described. Lesions of the recurrent nerve have frequently been ascertained to exist in such cases, but I am not aware of any observations having been made upon the change produced on the muscles of the larynx, or, in fact, of the symptoms having been traced to atrophy of these muscles.

"M. Legroux has given a truthful and graphic description of the effects of pressure upon the recurrent. To paralysis of the muscles of the corresponding side, producing subsidence of the aryteno-epiglottidean folds, he ascribes the imperfect aphonia and the 'frôlement,' or peculiar laryngeal blowing; but it does not appear that he had made any investigation into the state of the muscles themselves.

"At a meeting of the Pathological Society during the last medical session, Professor R. W. Smith presented a beautiful example of atrophy of the laryngeal muscles at one side in the larynx of a horse; and he informed the Society that Mr. Ferguson, from whom he had received the preparation, was of opinion that this state of the larynx was the pathological condition productive of what is termed 'roaring' in the horse.

"In making this communication Dr. Smith alluded to a drawing of Cruveilhier's, showing precisely the same lesion of the muscles, but with which there was no history connected. Dr. Smith observed upon the fact that the laryngeal muscles had not been hitherto accurately examined in cases of aneurism with pressure upon the recurrent.

"It is obvious, from the history of this case, that at its advanced stage it would be a matter of extreme difficulty, if indeed it were possible, to arrive at a correct diagnosis of the disease; the universal dulness of the left side, and the absence of the signs which with such certainty indicated the presence of aneurism at an early period, will sufficiently explain the probability of error. The extensive dulness here arose from the combined effects of the aneurismal tumor, of solidification of the lung, and of a partial empyema; but in other instances the occurrence of serous effusion into the pleura, the result of obstructed venous circulation, will tend to render a case obscure, which, if seen before this complication arose, would probably present all the ordinary signs of thoracic aneurism. Of the latter I have lately had an example in a case which I brought forward at a recent meeting of the Pathological Society.

"The practical deduction which I desire to make, then, is the following:

"That in cases of thoracic diseases, which are obscure owing to the supervention of other affections, we may be led to arrive at a correct knowledge of their true nature, and, with a high degree of probability, predicate the presence of aneurism, if the peculiar kind of aphonia, or, more properly speaking, extreme difficulty of raising the voice, with laryngeal blowing, or 'roaring,' be found to exist."

A vast fund of instructive matter may be derived from well reported autopsies of cases that were obscure in their phenomena during life, and they demand the careful study of physicians. It is a rare occurrence to find as interesting a case as the one quoted from Dr. Banks' Clinical Reports, and it will well repay the true student for any time he may devote to its study.

*A new method of reducing Dislocation of the Femur on the Dorsum Ilii.*—Dr. W. W. Reid, of Rochester, New York, read before the Monroe county Medical Society, at its annual meeting, in 1850, an essay of great value, on the reduction of dislocation of the Femur on the Dorsum Ilii. This is a subject on which the writer has spent a great deal of time, and he cheerfully acknowledges the importance of the contribution Dr. Reid has made to surgical art. After a series of excellent preliminary remarks, Dr. Reid developes his plan of reducing the form of dislocation of the femur referred to, without any mechanical contrivance whatever. This plan is the result of very accurate knowledge on the subject, and is detailed in the report of one of the three cases, reported by Dr. Reid.

The dislocation in Dr. Reid's case was four days old, and the patient, a strong, robust Irish woman, was bled freely and placed under the influence of tart. antimony. She was then placed on a firm table "about fourteen inches high, and about twenty inches wide, which gave the opportunity of throwing the whole weight of my body on the flexed limb, if I wished, while it gave me perfect command and control over it in every way. The patient was placed on her back, and a sheet folded lengthwise thrown across the upper edges of the pelvis bones, and each end given to an assistant, for the purpose of fixing the pelvis. Placing myself on the right and injured side, I seized the knee with my left hand, and the ankle with my right; I then flexed the leg upon the thigh; at the same time, slowly carried the knee and dislocated femur, over the sound one, pressing it firmly down upon it—and upward over the pelvis, constantly pressing it close to the body, moving it upward with a circular sweep over the abdomen, till the thigh was in a line with the right side of the body and the knee, pointing towards the right axilla. While the thigh was being carried up to this position, the bone or axis of the femur, was performing a kind of rotation on itself, whereby the toe was coming more outward and the heel more inward. In other words, as the knee went upward, the *obturator externus*, *quadratus*, &c., drew the head of the bone downward, and inward towards its socket. When the knee and thigh were in the position above indicated, the heel was strongly rotated inward, the knee drawn outward, and the foot carried across the thigh of the sound side, when the head slipped into its place, and the limb glided gently down into its natural position. In doing all this, comparatively very little force was employed, and very little pain produced, for the obvious reason, that, by this evolution, the muscles that were in a state of extreme tension and irritation by the displaced bone, were gradually relieved and relaxed, as the head of the bone descended and approximated its proper place, which it did by the action of these same extended muscles.

"It will be perceived that by this mode of operating, we make a *lever* of the shaft or bone of the femur, and a fulcrum of the edge of the pelvis—and by this means lift or dislodge the head of the bone,—while the abductor muscles draw it downward and inward, making it as it were, *back into* its place, through the rent of the capsular ligament. Whereas, if it were drawn by direct force, as by the pulley, the head and neck of the bone would act as a kind of hook, and would tear away the capsular ligament, if it were only slit, and as I believe it often, if not always, does tear off the tendon of the *pyriformis*, as I shall endeavor to show presently; for the *abductor* muscles are so strained, and hold the head of the bone so firmly to the dorsum, behind the ridge of the *acetabulum*, that it is next to impossible for it to mount over this ridge and into the socket, and must therefore descend behind it tearing every thing before it—ligaments, muscles and all—and hence the immense power required to reduce it by these means, and hence, too, the failures, the fractures of the neck, and the cripples, that have been made for life, by this barbarous and unscientific mode of reduction."

Dr. Reid deserves a great deal of credit for the ingenious developement he has made, in the essay under notice, of his plan of reducing dislocation of the Femur on the Dorsum Ilii. An anonymous writer in a recent number of the *Boston Med. and Surg. Journal* attempts to deprive Dr. Reid of his claim to the discovery, and singularly omits all reference to the fact that Dr. Reid disposes in advance of the futile claim set up for

Professor Nathan Smith, of New Haven. When the anonymous critic of the Boston Journal meets and refutes the courteous and cogent statements made by Dr. Reid, in reference to Professor Smith's plan of reducing dislocation of the femur, it will be time enough for the friends of Dr. Reid to join issue. Dr. Reid's statements are conclusive, and the attempt to do him anonymous injustice can do him no injury. When a man resorts to the anonymous to perpetrate an act of meanness which he would not do under his proper name, he shows that he has a feeling of shame in his wrong-doing, but has not firmness enough to resist his propensities.

In the Buffalo Medical Journal, for October, Dr. Reid's Essay having appeared in the August number of that Journal, Dr. Barnard, of Adrian, Michigan, reports a case of dislocation of the femur on the Dorsum of the Illium, in which resort was made to Dr. Reid's manipulations, and success crowned the efforts in thirty seconds.

*Ovarian Irritation.*—Dr. Fleetwood Churchill recently read, before the Association of the College of Physicians of Ireland, a practical paper on Ovarian Irritation. Dr. Churchill thinks it a distinct disease from that described by Dr. Tilt, under the name of subacute ovaritis.

Dr. Churchill says: "the chief characteristic symptom is an uneasiness, amounting in the greater number of cases to pain, and in some cases to very severe pain, in one or both iliac or inguinal regions, but most frequently in the left, which Professor Simpson seems to think is owing to the propinquity of the left ovary to the rectum, and the exposure to any irritation thence arising. This pain may be a constant dull aching, or it may be acute and occurring in paroxysms; it is greatly aggravated by standing, and generally by walking, indeed, in the severer cases, I have known the patient quite unable to walk.

"There is generally some complaint of fulness about the iliac region, but upon careful examination I have rarely been able to satisfy myself that this was more than a sensation; I certainly never felt anything like a distinct tumor. There is, however, always considerable tenderness, which in some cases is extreme to the slightest touch. When the irritation is great, it may be extended to the bladder, giving rise to a desire to evacuate its contents frequently, and causing great pain in doing so. Hysterical paroxysms are by no means unfrequent. In two of the most violent cases of hysteria that I have seen for some time, there was extreme tenderness of the region of the left ovary, and pressure there aggravated the hysterical paroxysm."

On the subject of treatment, Dr. Churchill says:

"I shall not enter at any length into details of the *treatment* of this disease, inasmuch as I have only my own experience to which I can refer. The choice of remedies will be governed, to a certain extent, by the health, strength, and state of constitution of our patient. With strong, healthy women I have tried leeches to the ovarian region, with some benefit but not complete success, nor in all cases; from six to twelve may be applied at once, and repeated, if necessary, after an interval. Poultices after the leeching are of use; and indeed, when no leeches have been applied, I have seen much comfort and relief derived from repeated poulticing. With delicate women, and they are frequently the subjects of this disease, bleeding in any form has appeared to me rather injurious than beneficial.

"I have tried the repeated application of small blisters with better results than leeching. The irritation of the surface certainly relieves the pain in many cases, and, if continued, may finally cure it; but I must confess I have seen it fail repeatedly.

"Anodyne liniments and anodyne plasters occasionally seem to afford relief, but they are often of little or no use; I tried anodyne enemata several times with partial success.

"In two or three cases I used the tincture of aconite, applied liberally to the iliac region, but I confess the result disappointed the expectations I had formed.

"Having failed in affording any relief in two or three obstinate cases, I determined to try the effect of opium applied to the upper part of the vaginal surface. I accordingly ordered some balls or pessaries to be made, somewhat in the mode of Dr. Simpson's medicated pessaries, each ball to contain two grains of opium, half a drachm of white wax, and a drachm and a half of lard. The whole, when mixed together, formed a ball about the size of a large marble, and I placed it at the upper end of the vagina by means of the speculum, leaving the patient in bed for the rest of the day. The success was quite beyond my expectation; the relief was very speedy, and in most instances complete. Even when the pain did return after a few days, a second application removed it. The tenderness disappeared with the pain, and no unpleasant consequences have resulted in any instance.

"I have now tried this remedy in a considerable number of cases, and with almost invariable success. I have rarely found it necessary to bleed or blister since I first adopted this plan; and I recommend it, with considerable confidence, to the profession. I may add that I have tried these pessaries in cases of dysmenorrhea, applying one the day before the catamenia were expected, with decided benefit.

"It is hardly necessary to say that, in this disease, the bowels should be regulated, and gently freed by medicine when necessary. If the appetite is bad, vegetable bitters may be given, and I have generally found it useful to combine some alkali with them."

*Early Viability.*—The following remarkable case is reported in the *Gazette des Hôpitaux.* It may afford a useful hint in cases of abortion:

"A lady, aged thirty-five, was confined, two months and seventeen days after having been driven over a rough road in a badly hung vehicle, of a very weak and small child; the whole extent of the gestation, calculated under circumstances very favorable to accuracy, had been exactly six months and ten days. The skin of the child was hardly formed of consolidation; there was no hair; the toes looked like a row of small pearls at the extremity of each foot, the fingers were so small that they were compared to lucifer matches; in fact, Dr. Ducos hardly thought the child could live.

"As it seemed, however, inclined to swallow, toast-and water mixed with milk was given. For the first ten days only, two tablespoonfuls of this fluid were given daily, the child always kept close to the fire. When two days old the infant took the breast, but it stopped at every second act of suction, and these were so feeble that the mother hardly perceived them. She was therefore obliged to procure another child to empty the breast. In this weak state the little creature continued until the time which would have been the natural term of gestation, without increasing much in size, but a little in weight. Six weeks after this time it began now and then to smile, being then full four months old, and has since been thriving satisfactorily."

*Cannabis Indica as a substitute for Ergot.*—The superior value of Cannabis Indica, over Ergot, as an excitant of uterine contractions, is a matter of reasonable doubt, but Dr. Christison is high authority on such subjects, and if his observations are confirmed in practice, the Indian hemp will become an important agent in obstetrical art.

The *Medical Examiner* says: "Dr. Christison, of Edinburgh, considers Indian Hemp, (Cannabis Indica,) to possess a remarkable power of increasing the force of uterine contraction during labor. He reports, in the August number of the *Edinburgh Journal of Medical Science*, some cases in which it was given, with this view, at the Maternity Hospital of Edinburgh. As compared with the action of ergot, that of Indian hemp presents the following points of difference: 'First,—While the effect of ergot does not come on for some considerable time, that of hemp, if it is to appear, is observed within two or three minutes. Secondly,—The action of ergot is of a lasting character, that of hemp is confined to a few pains shortly after its administration. Thirdly,—The action of hemp is more energetic, and perhaps more certainly induced, than that of ergot.' "

*Vesico-Vaginal Fistula, cured by an Operation.* By Prof. PANCOAST.—The October number of the Medical Examiner contains a report of an exceedingly interesting operation performed by Professor Pancoast. The subject was a lady who had been delivered, in 1848, of a child by mutilating instruments. After her recovery from her fatigue and exhaustion, she discovered that she had constant *stillicidium urinæ*, with all its attendant afflictions. The cause of the distress "depended upon a laceration affecting the urethra, so nigh to the neck of the bladder as to prevent the *sphincter vesicæ* from controlling the escape of the urine."

Professor Pancoast found it difficult, after various efforts, "to bring the injured part within the reach of his instruments, and he urged the patient to postpone the operation some months. In order to make the case more tractable for an operation, Professor Meigs prepared a peculiarly shaped canula, with a small funnel adapted to one end of a spindle, and a perforated disc at the other end. Professor Meigs, who reports the case, says:

"Such an instrument placed in the urethra, would not be thrust forth except the patient should make violent efforts to expel it. We hoped that by thus preventing the continual flow of the urine through the lacerated part, it might tend to close up so much as to render an operation practicable and successful; and I explained to her that such a conductor for the urine, if it could be worn for a long time, would perhaps serve at last as a cure, without resort to operation by the knife. She was begged to persist in the trial for several months—but came back again in about 150 days—being impatient of her sufferings.

"We thought great good was effected by the use of the canula, which she used during the greater part of the time, being, however, during her absence from town, annoyed to that degree, as to be sometimes obliged to suspend the use of it—which was reasonably to be expected.

"On a re-examination of the fistula in connection with Dr. Pancoast, on 13th of August, we found it much more easily reached with instruments than on the former visit of the patient, and the performance of the operation for its cure was determined on. On the 16th day of August, Dr. P., assisted by Mr. C. Neff, and by myself, the patient being on her knees upon a bed, proceeded to the operation. The callous margin of the fistula was first pared away—the mucous membrane on the right side of the wound was then seized by Dr. P. with a pair of delicate hooked forceps, and detached with scissors curved on the flat, for the space of 5-8ths of an inch around the semi-circumference of the orifice. The next stage of the operation was to obtain a corresponding surface on the opposite or further side, to overleap that already made. This was gained by Dr. P. in the following mode. The right lip of the orifice was grasped superficially with the forceps, and split mid-way between the two mucous membranes, to the depth of half an inch, with a lance-shaped knife curved on the flat, making, as it were, two layers of the vesico-vaginal septum, for the space of nearly an inch in length and half an inch in depth."

Professor Meigs pays a well-deserved compliment to Dr. Sims of Montgomery, Ala., in connection with this operation. The complimentary remarks contain an explanation of the suggestion made by Dr. Sims, and for that reason, they are quoted at length. Professor Meigs says:

"In a conversation with him in the end of July, 1851, he informed me of his many successful operations in vesico-vaginal fistula—a success which in my humble opinion entitles him to the praise and gratitude of our whole profession, which is always advanced by the ingenious and rational improvements made by such individuals, and which constitute a real progress of the Art. Dr. Sims communicated to me the fact, that if the patient, laboring under this accident, be placed in a certain position, the injured parts may be readily brought not only in sight, but so exposed as to render them easily accessible to the knife; the scissors, &c. &c.

"His own judgment prompted the discovery of this method, which consists in placing the patient on her knees with the ossa femoris standing in a vertical position while the summit of the thorax touches the table or bed on which she is placed.

"If such a posture be maintained for a few minutes, the fall of the abdominal and pelvic viscera downward toward the diaphragm, allows the os externum to relax, to open, and at last to offer so wide an aperture, that the cervix uteri may be clearly seen as in a metroscope. In the meantime, the fall of the viscera continuing, the walls of the vagina, to avoid the tendency to vacuum caused by the retreat of the pelvic contents toward the diaphragm, tend to expand, and like a balloon leave a spherical or orbicular cavity, as if the vagina was filled with a child's head, whereas it is filled with air.

"This discovery is in the highest degree applicable to the uses of curative surgery in vesico-vaginal fistula.

"The great experience and success of Dr. Sims will enable him, it is hoped, soon to give his results to the profession. In the meantime, I trust I shall give no offence while in describing the several steps of the operation on my patient, if I take this early occasion to thank Dr. Sims for his information, and to acknowledge how much I am his debtor therefor.

"From the moment of completing the operation above described, not a drop of urine escaped through the fissure. She wore the spindle-shaped canula during the ten or twelve days following and then removed it. The ligatures were easily cut away and picked out with dressing forceps, and we had the happiness to see the patient completely relieved of the effects of an accident, which nothing but a religious resignation could have enabled her to bear with patience, or to regard life otherwise than as a distressing burthen, to be borne only because it was God's will that she should patiently endure its weight. The cure was rapid and complete."

*Malignant Pustule or Carbuncle.*—In the first of these notes of the practical matter of various Medical Journals, some notice was taken of an epidemic exanthem described by Dr. Laycock. Since these remarks were written, I have met with a paper, by Dr. J. H. Baldridge, of Franklin, La., describing an epidemic carbuncle, which prevailed last July, in the parishes of St. Mary and Vermilion. Dr. Laycock was impressed with the belief that the epidemic he described was epizootic in its origin, and Dr. Baldridge seems to entertain no doubt that the epidemic he describes had a similar origin. In reference to the cause of the epidemic, Dr. Baldridge says: "Another attributes it to a poisonous marsh malaria. The first appearance of this disease here was among the mules which were in a healthy part of the parish, and we have had no excess of disease in the human subject generally attributed to malaria. I think the most plausible speculation to be the elimination of a powerful poison produced from the rapid decay of animal matter, caused by great heat and the absence of moisture, which being imbibed by the common carrion fly is reproduced in the living animal upon the principle of inoculation from the bite. Be this as it may, it is an established fact that the disease is reproduced by the bite of a fly, by contact with the secretions, blood and excrements of the animal affected. Laceration of the flesh with the hides of cattle which have died of this affection has been known to reproduce the disease.

"Those who treat stock affected with charbon, such as butchers, shepherds, tanners, and stockdrivers, are most liable to it. It generally appears upon the parts that are habitually exposed, upon the extremities, face, neck and breast."

*Treatment of Urticaria by the Sulphate of Quinine.*—The able editor of the *New Orleans Medical and Surgical Journal* bears the following testimony to the efficacy of quinine in *Urticaria.* The writer has often resorted to the use of that medicine in urticaria, and it has never disappointed the just expectations of its powers. Dr. Hester says:

"This is an eruptive disease, usually distinguished by elevations of the cuticle in the form of *wheals;* it is sometimes exceedingly obstinate, resisting all the means that may be brought to bear against it. We are induced to notice this affection, because recently we have met with two or three cases that yielded only to large doses of quinine.

"It is often quite simple in its nature, yielding readily to tepid baths, mild cathartics, and a restricted diet; but again, it is accompanied with much febrile disturbance, pain in the epigastrium, nausea, fulness in the head, and a burning sensation over the surface of the body; the face, hands, and feet swell; the eyes are almost closed; the tongue is loaded with a white coat, and the itching is intolerable at times. Again, the eruption is accompanied with severe articular pains, all of which phenomena serve to complicate the exanthema, and augment the difficulties of the case. Dr. Wickham and M. Legrouse, of the Hospital Beaujon, report some cases of the worst forms of Urticaria, which were promptly cured by full doses of quinine, continued for two or three days.

"Treated with quinine the articular pains, the painful tumefaction of the face, feet, and hands, the eruption itself, rapidly disappeared, together with the nausea; febrile excitement, and indeed all the distressing symptoms."

*Pertussis.*—This disease is often intractable under the ordinary forms of treatment, and the management of intractable cases often degenerates into empirical methods. In the *Stethoscope*, for October, Dr. Madison, of Petersburg, Va., pleads for a novel pathology, and there is but little reason to doubt the truth of his views. Dr. Madison thus explains his pathology and treatment:

"Now, a careful investigation of a good many cases, which have recently presented themselves to my observation, induces me to believe that the cough is essentially *nervous* in its character, developed by a specific irritation of the *spinal cord* extending from the origin of the *eighth* pair down to that of the *phrenic* nerve. This conclusion is arrived at by a species of negative induction. If the cough were dependent upon pneumonic inflammation, we should have all the physical signs of pneumonia constantly developed. If it originated in a bronchitic irritation, we should have hooping cough with every attack of bronchitis. If irritation of the *peripheral* extremity of the pneumogastric nerve were its cause that in itself would be sufficient to produce pneumonia in every case! Inasmuch therefore as these causes seem evidently inadequate to develop the characteristic phenomena of hooping cough, and since these phenomena are clearly of a nervous character, and the parts involved in the disease are only those to which the eighth pair and phrenic nerves are distributed, I am forced to believe that the source of irritation—the cause of the disease—is to be found in the spinal cord at the roots of these nerves.

"Acting in accordance with this belief, I have adopted a method of treatment remarkably simple and eminently successful. It consists in the application of a *blister to the nucha*, which, upon the principle of counter-irritation, speedily and permanently relieves all the distressing symptoms of the cough. In a large majority of cases a single application of the blister will suffice for an instantaneous cure. Those which resist the first application yield to a second, aided by the internal administration of quinine, or of iron combined with quinine in the form of the citrate. I would not recommend the use of the blister in case of infants of a very tender age; but pustulation by means of the unguentum antimonii or the ol. tigli will accomplish the desired object.

"Now, whether these brief views in regard to the 'pathology of pertussis,' be correct or not—whether they be condemned as theoretical, and the treatment as empirical—certainly the success which has attended my efforts, justifies me in warmly recommending it to the attentive consideration of my professional brethren."

*Prophylaxis of Puerperal Fever.*—Professor Mettauer, of Randolph Macon College, Va., appears in the October No. of the *Stethoscope* in defence of the views he published some months ago, on the prophylaxis of puerperal fever, and he certainly sustains his views in a triumphant and most satisfactory manner. The writer has long been familiar with the practice, which Professor Mettauer has expounded with force and clearness. In 1833 I published a paper in the Transylvania Journal of Medicine, in which I endeavored to elucidate the very principale which professor M. has placed upon invulnerable ground. My remarks were upon Puerperal Mania, and I then had to meet the very error which Dr. G. A. Wilson, of Richmond, Va., has thrown across the path of Professor Mettauer. In the paper referred to, published in 1833, I used the following language:

"The error to which I allude, is his opinion of the proximate cause of the affection, which, he says, 'is neither congestion nor inflammation, but one of excitement without power.' This is John Hunter's definition of irritation, clothed in a livery of new words, and like it, is both unmeaning and unsatisfactory. But passing over its obscurity, I deny the truth of the position. Nothing can be more conclusive on the subject than Dr. Gooch's own testimony, given in the form of observations, and a case related by the Doctor as having occurred in his own practice. He says: 'the alimentary canal is generally disordered in its secretions—the symptoms which indicate this, are a furred tongue, an offensive breath, and above all, dark and offensive stools.' The case alluded to above confirms this. 'Her pulse was soft, and never very quick, face pale, conjunctiva yellow, tongue furred, and her bowels costive. In fifteen months afterwards, she was confined again, without any recurrence of the disease; *about a week before this latter delivery she had the jaundice, of which she was cured by calomel and aloetic purgatives before she fell into labor.*'

"Can any state of the system be better marked by symptoms, than congestion was in this case? And in every one reported by Dr. Gooch, where the symptoms are specified, the same results obtain; and yet puerperal mania is caused by 'excitement without power!' Our author thinks himself that the most important inquiry in this disease as in every other, is in reference to "the morbid state of organization on which the disorder of the mind depends,' and I fully coincide in the opinion. If uncertain on this point, we have no guide to the treatment, and if in error in the pathology, the chances are two to one for the same in remedial measures.

"But to return to the question of its proximate cause. What other satisfactory account can be given of vitiated secretions, than their origin in a disturbed state of the circulation, or in other words, an accumulation of blood in some of the secreting organs and a diminution of it in others? None. Step by step, this proposition has been already established in the preceding numbers of this Journal, beyond the reach of a reasonable cavil. And when we see the bold and energetic practitioner laying hold of the principle, as the foundation of his treatment, and find him speedily subduing the horrors of the disease and restoring a female to reason, a wife to her husband, and a mother to her family, it is no small argument in favor of the truth of the pathology."

A case of unusual interest in point, was reported, where the prophylactic plan, thus indicated, was forcibly shown. The female was the mother of three children, and had been the victim of violent puerperal mania in each case. The second attack lasted 10 months. Prior to her fourth confinement she was placed under the treatment of my then partner, and he placed her at once under preparatory mercurial purgatives. The attacks usually commenced between the third and fifth days, and between the second and fifth days of this fourth labor, she began to exhibit decided signs of puerperal mania. She was restless and sleepless at night, and had a sullen and fixed gaze. But by persevering in the treatment nightly, the attack was warded off. This was the only time this patient was placed under preparatory treatment, and it was the only time she ever escaped an attack of puerperal mania. Since that labor she has been confined some six or seven times, and in each of these she had violent puerperal mania.

I have often resorted to this plan of prophylaxis in similar cases, and in puerperal fever, with the same gratifying results, in every instance, as were attendant on Professor Mettauer's efforts. That gentleman deserves a great deal of praise for the able manner in which he has advocated the practical truth he urges upon the readers of the *Stethoscope*.

Dr. Mettauer thus disposes of the objections urged against his truths: "In reply to the third and fourth interrogatories of Dr. Wilson, that is, 'What good can result from throwing the whole abdominal viscera of a patient on the verge of peritoneal inflammation into violent commotion?'—and 'Who would prescribe active exercise for a knee-joint in danger of inflammation?' I will remark in the first place, that the idea that a cathartic throws the whole abdominal viscera into violent commotion is entirely gratuitous and unfounded, unless agents are employed of violently irritating and drastic qualities, which no practitioner of judgment and prudence would be likely to do in puerperal cases; but the reverse is the case in many diseases of these organs, as it tranquilizes and composes them when they are already disquieted. Would any judicious practitioner be unwilling to purge patients laboring under the abdominal disturbances consequent on colic, after the spasms have subsided, for fear of harassing them in their fatigued condition, and thereby endangering them from inflammation? Would Dr. Wilson himself withhold purging under such circumstances, for fear of superinducing inflammation in those organs? And yet he would inculcate the paradoxical doctrine, not to purge after delivery, for fear of perturbing the fatigued organs concerned in pregnancy and labor, while he would advise an immediate resort to it after the subsidence of the spasm and pain of colic, to prevent inflammation in the intestines, and in conditions of the organs in both cases very nearly identical. I would remark also, in response to this interrogatory, that I have conclusively shewn by my sixty-two cases, that the idea that purging harrasses the organs concerned in pregnancy and labor after delivery is entirely gratuitous and unsupported, as in not one of them did the patient complain of the slightest harassing or ill effect from the purging plan: and I think the sixty-two cases also furnish very strong presumptive evidence of the beneficial effects of purging in the prophylaxis of puerperal fever, not however by 'throwing the whole abdominal viscera of a patient on the verge of peritoneal inflammation into violent commotion,' but by restoring to the organs concerned in pregnancy and parturition their normal physiological actions. Certainly no one in his senses would 'prescribe active exercise for a knee-joint in danger of inflammation,' because experience has abundantly shewn that it is improper, while that excellent *teacher* has also demonstrated, in numerous instances, that the peristaltic action of the intestines can be safely and beneficially excited by cathartics in the treatment of enteritis. Besides, the intestines are not always involved to any great extent in puerperal inflammation, but the disease is confined to the uterus and its appendages, the parietic peritoneum, bladder, &c. And in the inflammations of these organs cathartics are our best remedies after venesection, both as prophylactic and curative measures; and on these organs purging could hardly exert a perturbing influence; and if Dr. Wilson's views are correct, perhaps the sixty-two cases were of this description, as the treatment did not harass the patients nor induce puerperal fever, as seemed to be the result of purging in his cases."

Professor Mettauer has truth on his side; it is sustained by facts gathered from an enlarged experience, and his doctrines on the prophylaxis of puerperal fever, cannot fail to find a permanent lodgement in the minds of all physicians who submit them to the tests of use and observation.

